# How In Vivo Alteration of Hip Replacement Wear Mode Can Cause a Voluminous Inflammatory Reaction and an Excessive Titanium Exposure

**DOI:** 10.3390/jcm14010210

**Published:** 2025-01-02

**Authors:** Luca Sutter, Deborah J. Hall, Lydia Bischoff, Corina Dommann-Scherrer, Michel Schläppi, Robin Pourzal, Nadim Hallab, Christoph Meier, Peter Wahl

**Affiliations:** 1 Division of Orthopaedics and Traumatology, Cantonal Hospital Winterthur, 8401 Winterthur, Switzerland; luca.sutter@uzh.ch (L.S.); lydia.bischoff@ksw.ch (L.B.); michel.schlaeppi@ksw.ch (M.S.); christoph.meier@ksw.ch (C.M.); 2 Department of Orthopedic Surgery, Rush University Medical Center, Chicago, IL 60612, USA; deborah_hall@rush.edu (D.J.H.); robin_pourzal@rush.edu (R.P.); 3 Institute of Pathology, Cantonal Hospital Winterthur, 8401 Winterthur, Switzerland; corina.dommann@ksw.ch; 4 Department of Immunity and Emerging Pathogens, Rush University Medical Center, Chicago, IL 60612, USA; nadim_hallab@rush.edu; 5 Faculty of Medicine, University of Bern, 3010 Bern, Switzerland

**Keywords:** arthroplasty, wear, adverse reaction to metal debris, ARMD, titanium, toxicity

## Abstract

**Background:** Wear particle reaction is present in every arthroplasty. Sometimes, this reaction may lead to formation of large pseudotumors. As illustrated in this case, the volume of the reaction may be out of proportion to the volume of the wear scar. This case also is the first description of elimination kinetics of systemic titanium exposure caused by wear of a hip arthroplasty. **Methods:** Case report. **Results**: A 85-year-old male required revision after total hip arthroplasty due to aseptic loosening of the cup. A massive local adverse reaction to metal and polyethylene debris developed before revision, much larger than the implant damage would intuitively suggest. In this case, in vivo transition in wear mode from edge loading to impingement wear resulted in excessive titanium and polyethylene wear and subsequently a voluminous macrophage reaction and an excessive systemic titanium exposure, with blood concentrations showing a very long elimination half-life of more than two years. **Conclusions:** The volume of the wear particle reaction is dictated by the volume of the inflammatory cells, not of the wear particles. To the best of our knowledge, this is the first description of elimination kinetics in case of systemic titanium exposure. While the tissue response is caused by a sudden increase of titanium and polyethylene debris, titanium is detectable through whole blood, not serum, analysis and thus be an indicator for risk of failure due to abnormal articulation of the joint replacement. Such measurement may be useful if changes in implant position are detected radiographically. Major elevations of titanium concentrations may require revision, as for any other metal ions.

## 1. Introduction

Metallic implants are commonly used in joint replacement and fracture fixation. Wear is a common issue, particularly on the articulating surfaces of mobile implants [1]. It may also develop after loosening or breakage of static implants, i.e., failure of fracture fixation, corrosion of modular connections or rupture of joint replacement components [2]. Fractures may also affect monobloc stems, not only modular implants [3,4]. Accumulation of metal ions and particles with a consecutive local adverse reaction to metal debris (ARMD), or development of systemic toxicity, would be common complications, particularly following wear from metal-on-metal (MoM) bearings in total hip arthroplasty (THA) [5]. Regarding systemic toxicity of metal ions, particularly cobalt (Co) toxicity has been documented in multiple cases [6,7,8]. Other metals may however also cause local or systemic toxicity issues [9,10,11].

The purpose of this report is to illustrate how a wear defect of a metallic implant may cause a voluminous inflammatory reaction, as there seemingly is a major discrepancy between both the volume of material loss and the volume of the observed ARMD. This can well be illustrated by this case of a MoM THA, revised due to loosening as well as due to a massive local ARMD. Particularly as this case provides exceptional and new insights into systemic titanium exposure. How metal ion concentrations should be measured and interpreted is also reviewed.

## 2. Case Description

**Figure 1:**
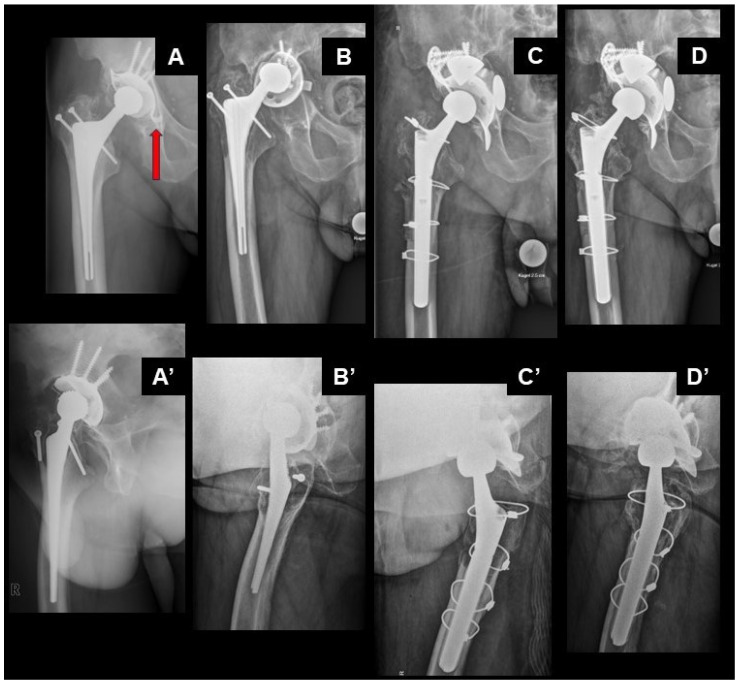
Zone of interest of anteroposterior radiographs of the pelvis showing the right hip (**A**–**D**), respectively corresponding faux-profile (**A’**) or axial radiographs (**B’**–**D’**). The oldest radiographs available are from 11 years after THA, respectively 9 years after the first revision of the cup, 13 years before the revision described (**A**,**A’**). Note the metal-polyethylene-sandwich cup and the small-diameter metal-on-metal bearing, as well as the improper placement of the reinforcement ring. The hook normally should engage the acetabular fossa to reduce the risk of dislocation (arrow). Dislocated hip implant 3 months before revision illustrated in (**B**,**B’**). Postoperative radiographs after revision are illustrated in (**C**,**C’**). Note successful reconstruction of the centre of rotation and of the leg length. Follow-up 3 years after revision in (**D**,**D’**).

THA had been performed on the right side 24 years earlier in a now 85-year-old male, using an uncemented Fitek cup with a 28 mm Metasul metal-polyethylene-sandwich bearing, and an uncemented BEO stem (Zimmer Biomet, Winterthur, Switzerland). The cup already had to be revised after 2 years due to delamination of the Sulmesh coating with consecutive loosening. Reconstruction had been performed using a reinforcement ring (Ganz ring, Zimmer Biomet) in combination with a cemented Mueller low-profile cup with a 28 mm Metasul bearing (Zimmer Biomet). Eighteen years after the first revision, aseptic loosening of the reinforcement ring was noticed (Figure 1), causing the cup to tilt and migrate over time. The cup had been correctly oriented initially, with an inclination of 32° and a radiologic anteversion of 19° (Figure 1A). However, the reinforcement ring may have lacked proper support, as the distal hook did not engage the distal end of the acetabular fossa and as the lateral flange was placed medially to the lateral wall of the acetabular roof (Figure 1A). Increasing symptoms finally lead to consultation in our department. A CT-scan confirmed major osteolysis of the acetabulum, but both columns of the acetabulum were still in continuity, despite being thinned out and despite a defect of the medial wall (Paprosky Type IIIb) [12]. No images are provided here as metal artefacts severely impaired quality and readability of single pictures. Radiologically, the stem appeared well-fixed, despite osteolysis present in the trochanteric region. A fluid collection of approximately 5 cm diameter was present anteriorly to the joint. Preoperative joint aspiration showed haemorrhagic, but not metallotic fluid, with <50 leucocytes/µL, remaining sterile after prolonged incubation. Based on these findings and a moderately impaired general condition of the patient, it was decided to perform revision of the cup through an anterior approach, retaining the stem to permit full weight-bearing postoperatively.

**Figure 2:**
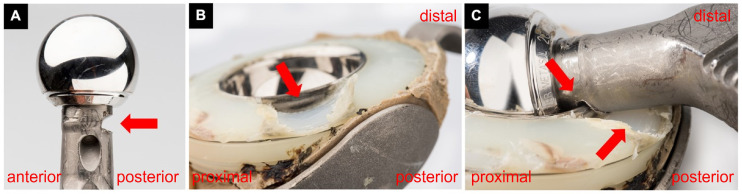
Macrophotographs of the retrievals. In (**A**) implant head and neck with a posteriorly situated wear defect, measuring 13 mm in width, 4.4 mm from proximal to distal, with a depth of 4.25 mm. Total volume loss thus measured approximately 162 mm^3^, considering the defect as a half-cylinder. In (**B**), illustration of the cup, showing a posterior wear defect. In (**C**), illustration of both the cup and the stem assembled, showing posterior component impingement. The defect zones align perfectly. The edge of the cobalt-chromium-alloy cup of the metal-polyethylene-sandwich cut into the softer titanium alloy of the stem during repetitive impingement, whereas the neck caused a defect of the softer polyethylene (red arrows).

**Figure 3:**
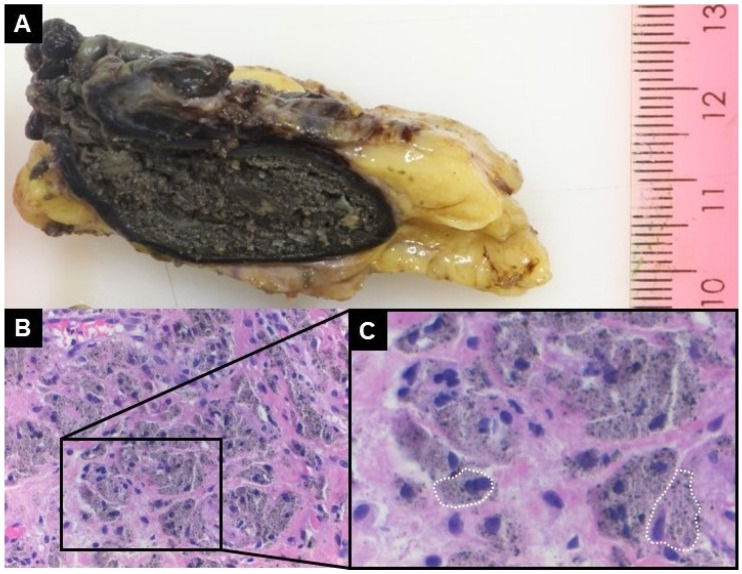
In (**A**), macrophotograph of the sectioned periarticular solid black necrotic tumor, which was located posteriorly to the hip joint, having a diameter of 3.8 cm after fixation, surrounded by adipose tissue. In (**B**,**C**), microphotograph at high magnification (hematoxylin and eosin staining ×200 in (**B**) and ×400 in (**C**), depicting proliferated macrophages showing basophilic stained oval nuclei and expanded cytoplasm, containing numerous phagocyted black metal microparticles (two macrophages delineated with a dotted white line).

At surgery, some decilitres of a pulpy, greyish fluid were evacuated from the joint. The neocapsule, the ARMD pseudotumor, and the osteolytic lesion were debrided. Multiple biopsies were sampled for microbiological and histological analysis. As a previously not identified indentation of the neck of the stem was observed (Figure 2), estimated to be critical regarding fatigue fracture risk based on size and location, as the head could not be removed and as exposure was insufficient for reconstruction of the acetabulum despite extension of the approach along the iliac crest, conversion to a transfemoral approach was performed. A significant, mostly solid ARMD pseudotumor (Figure 3A) was present posterolaterally to the hip joint, which had not been present on the CT-scan performed 3 months preoperatively, causing complete destruction of the external rotators. Reconstruction of the hip is illustrated in Figure 1. Intraoperative blood loss required transfusion of 2 red blood cell concentrates in addition to retransfusion of approximately 1000 mL of salvaged blood.

Postoperatively, a short observation in the intensive care unit was required. Wound edge necrosis of the anterior approach could be managed with wound dressing, requiring no reoperation. Further recovery was uneventful. All tissue samples for microbiologic workup remained sterile. Initially, partial weight-bearing had been prescribed for 8 weeks, due to the extended trochanteric osteotomy and reconstruction with an uncemented, distally fixated stem before proceeding to full weight bearing. Now 5 years after the revision, there remains a reduction of the range of motion (flexion/extension 85/10/0° and external/internal rotation 30/5/0°) and there is a leg length discrepancy of 10 mm (left shorter). Nevertheless, the patient is satisfied with the result and needs no adaptation of his shoes. He returned to his usual home and still lives in his old farmhouse, despite poor adaptation of the interior.

**Figure 4:**
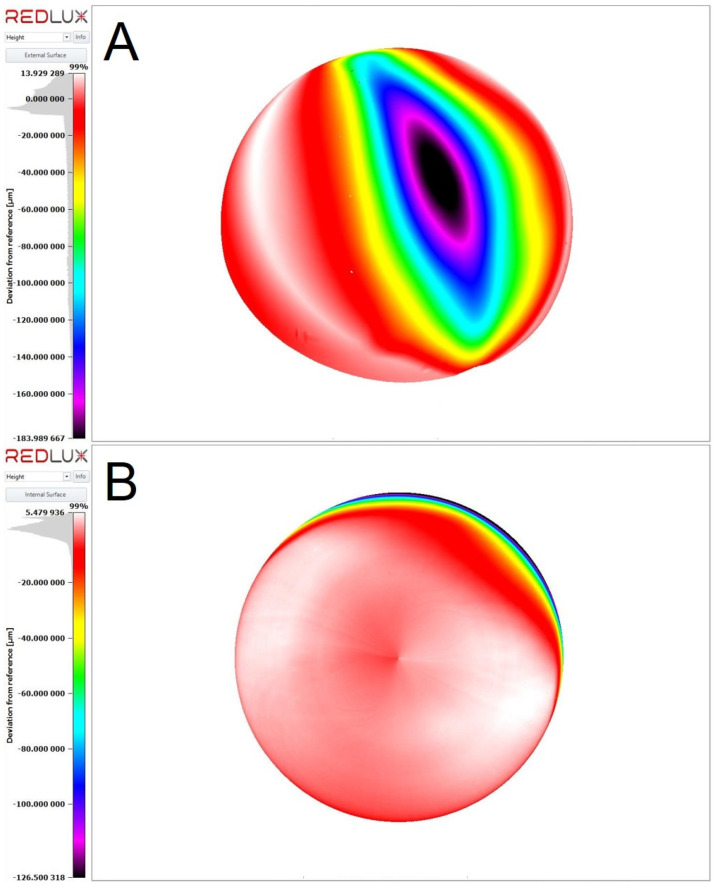
Heat maps showing bearing surface wear based on metrology data collected with an optical coordinate measuring machine (RedLux, Ortholux). (**A**) shows a narrow wear scar on the head’s bearing surface, which is characteristic for edge loading. The wear of the head amounted to 32.34 mm^3^. The wear scar of the metal liner is shown in (**B**), located at the rim and was associated with 8.02 mm^3^ of material loss. Total wear of the bearing surfaces amounts to 40.36 mm^3,^ which is only a fourth of the defect size of the neck of the implant.

Upon retrieval of the implant, component impingement (wear mode 4A [13]) was obvious, resulting in severe wear of the Ti-alloy stem articulating against the rim of the CoCr-polyethylene-sandwich liner (Figure 2). Assuming a cylindrical shape, the resulting material loss on the stem may be estimated to be ~162 mm^3^. Wear of the bearing surfaces as well as of the head/neck taper was quantified with a 5-axis optical coordinate measuring machine (CMM) (OrthoLux, RedLux, Romsey, UK), as described elsewhere [14]. The wear of the head amounted to 32.34 mm^3^, while wear of the metal liner was 8.02 mm^3^, summing up to 40.36 mm^3^ (Figure 4). The wear scar shape and location on both the head and cup were characteristic of edge loading or microseparation (wear mode 2 [13]). There was no damage at the taper interface.

**Figure 5:**
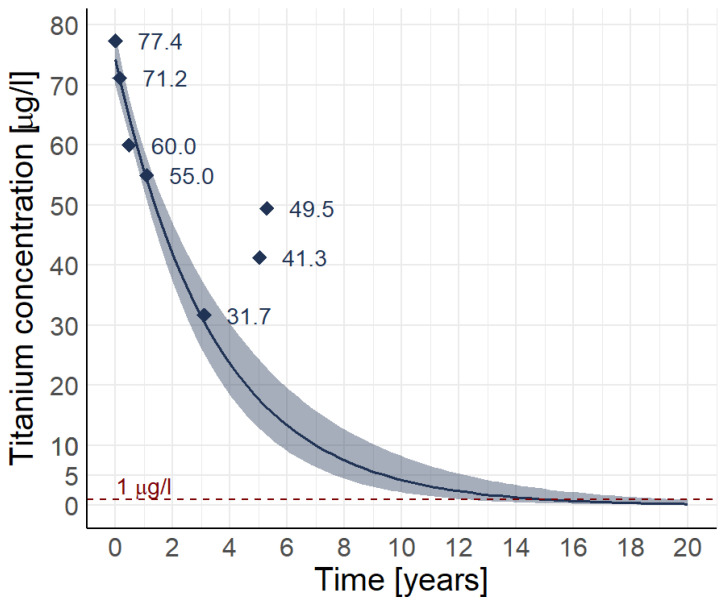
Graphical illustration of the titanium concentrations in whole blood over time observed in the patient described. Expected concentrations were extrapolated from the individual measurements, not considering the last two values, as an unexplained secondary increase was observed. The calculated elimination half-life of titanium in the blood in this patient thus was 2 years and 233 days. Without this secondary increase, normalisation of the titanium concentration would not have been expected before 14 years postoperatively. The dotted line at 1 µg/L marks the normal reference value.

Considering the voluminous ARMD observed, metal ion concentrations were measured both in the tissues sampled as well as in the blood. Chromium (Cr) was measured at 6.6 µg/L, Co at 18.9 µg/L and titanium (Ti) at 77.4 µg/L (all values indicated for whole blood). Aluminium (Al) was also measured due to the damage on the stem made of an aluminium-containing alloy, but blood concentrations were not elevated. In the tissues, Al concentration was however at 9400 µg/kg, Cr at 418,000 µg/kg, Co at 41,600 µg/kg and Ti at 406,100 µg/kg. The Ti blood levels slowly decreased over time (Figure 5). All concentrations normalised over time, except Ti, which was still elevated at the last follow-up examination 5 years postoperatively, respectively even increased again secondarily. Based on the concentrations measured over time, the elimination half-life from the blood of Ti in this patient was 2 years and 233 days, excluding the last two values. Of note, the patient presented a chronic renal disease KDIGO grade 3, with an estimated glomerular filtration rate initially around 45 mL/min/1.7 m^2^, whereas at 5 years follow-up it was between 30 and 35 mL/min/1.7 m^2^ [15].

**Figure 6:**
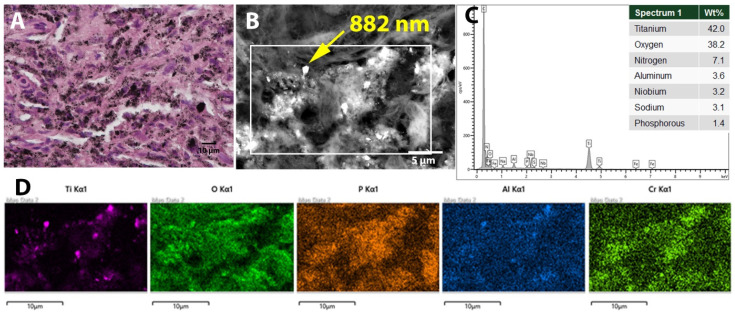
In (**A**), high magnification photomicrograph demonstrating dense metal debris within macrophages (haematoxylin and eosin, ×600). In (**B**), backscattered scanning electron micrograph of the same metal particle-containing area as in A (×4000). In (**C**), EDS spectrum of the bright 882 nm particle marked in (**B**) (yellow arrow), showing an elemental composition corresponding to the titanium alloy (TiAlNb) of the stem. In (**D**), EDS mapping of the area within the white box of (**B**), showing the location and composition of the particles seen in (**A**,**B**).

Tissue specimens retrieved from the neocapsule, the pseudotumor and the acetabular osteolysis were fixated in 4% buffered formalin for 24 h and processed for paraffin embedding. One-micrometer-thin tissue sections were stained with haematoxylin and eosin for histological examination. Histology showed that the periarticular black mass (Figure 3A) consisted of extensive necrosis containing black microparticular debris without birefringence in polarized light, identifying the material as non-ferrous metal particles [16]. The necrotic tumor was demarcated by a marked macrophage reaction demonstrating phagocytosis of the metal particles (Figure 3B,C), some bone sequesters and siderophages indicating older haemorrhage. Tissue samples of the neocapsule of the hip and of the acetabular osteolysis exhibited a marked macrophage and foreign body giant cell (FBGC) response to abundant metal and polyethylene debris, and to a lesser degree bone cement vacuoles. The amount of metal and polyethylene particles present within the macrophages contributed largely to the dominant dark-grey appearance of the tissue. Of note would be that no relevant lymphoplasmocytic or eosinophilic inflammatory reaction was present. In summary, the synovial lining integrity, the inflammatory cell infiltrates, and the tissue organization showed an ALVAL-score (aseptic lymphocytic vasculitis-associated lesion [17]) of 5 out of 10 points. Energy Dispersive X-ray Spectroscopy (EDS) analysis confirmed that the metal particles within the histiocytic cells were composed of Ti, Al, and niobium, constituent parts of the alloy of the stem. Particles containing Cr and phosphorous were also identified in the tissue, especially intracellularly (Figure 6).

## 3. Discussion

The described case presents exceptional features for discussion regarding planning of revision THA, development of a massive ARMD following unusual wear of implant components, including non-bearing surfaces, and systemic exposure to metal particles and ions.

Based on the shape, size, and location of the wear scar on the bearing surfaces, edge loading—or micro-separation—appears to have been the initially predominant wear mode [13]. In MoM THA, edge loading is associated with larger Co containing particles, while normal wear primarily releases Cr-oxide particles and Co ions at the articulation. The observed intracellular Cr-phosphate is consistent with phagocytosis of Co-containing particles, indicating Co ion release within the tissue rather than directly from the implant [14,18,19,20]. Despite abnormal CoCr wear, no significant lymphocyte-dominated adverse local tissue response was observed, as indicated by a relatively low ALVAL score [17]. This is an important finding indicating that Ti particles are more chemically stable (i.e., less biologically reactive) compared to other metal such as Co and Cr, producing minimal lymphocyte reactivity even with large amounts of metal debris, similar to polyethylene particles [21]. The wear mode changed over time to impingement wear, with articulation primarily taking place between the Ti-alloy stem and the edge of the CoCr socket, respectively the polyethylene of the sandwich cup (Figure 2) [13]. This change dramatically increased wear volume, predominantly driven by excessive release of Ti-alloy and polyethylene particles. The inflammatory reaction to Ti particles may possibly be a particular cause of voluminous ARMD pseudotumors, considering similar findings in another case report [11]. This process likely occurred over a relatively short time, leading to a severe inflammatory response characterized by particle-laden macrophages and tissue necrosis. Surprisingly, the ARMD pseudotumor was much larger intraoperatively than on the CT-scan performed just 3 months earlier, where no tumor located posteriorly to the joint was identifiable. This discrepancy suggests rapid ARMD development within a few months before revision due to sudden influx of Ti and polyethylene particles. To account for such rapid progression, shorter delays between the latest 3D imaging and revision may be advisable.

The volume of the inflammatory reaction appears disproportionately large to the size of the metal defect on the implants, even considering wear from the bearing surfaces. The notch on the neck of the stem may be estimated at 162 mm^3^ (Figure 2), whereas wear of the bearing surfaces was relatively low (40.36 mm^3^ overall) without measurable wear on the taper (Figure 5). Assuming a conservative average particle size of 0.5–1 µm (without nano particulate), this yields approximately 1 trillion particles capable of inducing a biological response. Combined with the elevated metal ion concentrations in the tissues, this reinforces the hypothesis that the inflammatory reaction primarily resulted from the notch on the neck of the stem and the associated polyethylene wear particle release. The volume of the pseudotumor, over 1000 times larger than the defect, is expected to induce a exuberant innate immune response, mostly defined by the phagocytic macrophage inflammatory cells, and not by the volume of the much smaller wear particles (Figure 3). While the metallic wear particles are typically <0.1 µm in size, macrophages have a diameter around 20 µm [22,23]. Details regarding the mechanisms of the immune response to metallic particles and their interaction with lymphocytes, macrophages, osteoblasts, and fibroblasts, are available in a recently published review [24]. This pathophysiological process explains the complications observed in this case, including pain, aseptic inflammation, soft-tissue damage, pseudotumor formation, as well as development of osteolysis and loosening. While small-diameter MoM THA have low long-term revision rates [25], the polyethylene-metal-sandwich liner design permits specific complications, such as impingement wear of the neck or dislocation of the metal cup, as reported in isolated cases [26,27].

Co and Cr concentrations were elevated in both blood and periarticular tissues, with massively higher levels in the latter. Ti concentrations were highest by far. While Co and Cr blood levels normalized within months, Ti blood levels remained elevated for years post-revision (Figure 5). This persistence likely reflects the large volume of Ti debris generated under Mode 2 wear within a short amount of time and its dissemination locally and systemically to tissues not accessible to surgical debridement. Evidence regarding Ti toxicity or other systemic effects in arthroplasty patients remains scarce [10,11,28]. Severe metal particle deposits have been described in various organs, yet only isolated serum and/or whole blood values are generally reported due to the challenges of fully isolating particulate debris from serum, whole blood or tissue for analysis [10,11]. Although serum analysis avoids dilution by red blood cell volume, its technical difficulties maintaining a metal free environment have led a more widespread adoption of whole blood for clinical diagnosis. However, in case of suspected loosening, evidence of high wear, Ti- associated osteolysis, pseudotumor formation, or skin metallosis, blood or serum Ti levels may aid in surgical decision making. Guidelines regarding Ti blood/serum level interpretation vary, but according to the Mayo clinic, circulating Ti is <1 µg/L in individuals without implants, 1.0–3.0 µg/L serum with a well-functioning prosthesis, and >10 µg/L serum suggests significant wear (Mayo Clinic Laboratories. Titanium, serum. https://www.mayocliniclabs.com/test-catalog/ClinicalþandþInterpretive/89367. Accessed 10 December 2024). Past reports have shown an average Ti serum level of 135 ug/L in a cohort of failing total knee implants [29]. In this case, the elimination half-life from the blood of Ti was notably slow, with 2 years and 233 days in the first 4 years of follow-up. At the latest follow-up, 5 years post-revision, blood Ti levels even had increased slightly.

As the slight increase could not be definitively explained, the last values were excluded from the half-life calculation. Another mechanism may have interfered. As the Ti blood levels are a result of release from the tissues and elimination via the kidneys, one possible explanation could be that the kidney function decreased slightly over the years. At revision, the estimated glomerular filtration rate was ~45 mL/min/1.7 m^2^, whereas at 5 years follow-up it was between 30 and 35 mL/min/1.7 m^2^. Increased patient activity after recovery, allowing him to manage his farmhouse independently again, may have also released additional Ti from the tissue. Alternatively, slow oxidation of the Ti particles could lead to late dissolution, whereas CoCr particles degrade much faster, particularly intracellularly. A measurement error is unlikely as levels were determined using mass spectrometry, with all assays conducted in the same laboratory.

The case highlights that, beyond Co and Cr, which are well-documented for their systemic toxicity from wear of MoM bearings in THA, other metals must also be considered [9,10]. Metal ion concentrations should be measured in whole blood, not in serum, as threshold definitions are based on whole blood, and as both values are not interchangeable as the intra- and extracellular compartments act independently, at least for Co and Cr ions [7,30,31]. Systemic effects of excessive Ti exposure remain poorly understood, and any toxicity mechanisms remain unknown, despite the extensive use of Ti-oxides in agriculture, as well as in the food and the cosmetics industry as a white pigment [10,11,32,33,34]. Measuring Ti concentrations faces many challenges, with graphite furnace atomic absorption spectroscopy (GF AAS) and quadrupole inductively-coupled plasma mass spectrometry often overestimating levels due to particulate interference and nano-particulate erroneously skewing interpretation of ion content [35]. This makes the use of Inductively Coupled Plasma—Mass Spectrometry necessary, limiting the availability of Ti assays in clinical practice [32]. Symptoms of metal toxicity, such as cognitive deficits, fatigue, depression, forgetfulness and dementia [10,11,24], may be difficult to recognize in elderly patients, who may suffer from cognitive impairment from other aetiologies or be limited by other neurologic comorbidities. In this case, the patient’s initial mild cognitive impairment was attributed to age, but, retrospectively, may well have been related to metal toxicity. This impairment likely contributed to a long delay between loosening of the cup and revision. Arthroprosthetic toxicity should be considered, alongside osteolysis and bone defect evolution, when managing such patients.

To the best of our knowledge, screening possibilities are very limited, particularly for elderly patients impaired for various reasons. Routine dementia tests may lack sensitivity to detect subtle cognitive deficits. More sensitive testing may be necessary but is not available routinely [36]. Usual mobility/frailty tests are impossible to perform in case of a major orthopaedic impairment, further limiting evaluation. More severe neurologic symptoms have been described in a recent case report with whole blood concentrations exceeding 100x the values observed in patients with well-functioning THA [11].

## Data Availability

Raw data available upon request.
